# New Insights into Cardiac Intensive Care

**DOI:** 10.31083/RCM49773

**Published:** 2026-07-24

**Authors:** Cheng Chen, Yanming Wu, Biao Wang

**Affiliations:** ^1^Department of Cardiology, Suzhou Ninth Hospital Affiliated to Soochow University, Suzhou Ninth People’s Hospital, 215000 Suzhou, Jiangsu, China; ^2^Department of Cardiology, Coronary Care Unit, Suzhou Ninth Hospital Affiliated to Soochow University, Suzhou Ninth People’s Hospital, 215000 Suzhou, Jiangsu, China

**Keywords:** cardiac intensive care, hemodynamic monitoring, mechanical circulatory support, artificial intelligence, precision therapy, prognostic evaluation

## Abstract

In recent years, there have been significant advances in both theoretical knowledge and technological applications in cardiac intensive care, a crucial area of cardiovascular medicine. This study methodically outlines the most recent developments in the field’s research, including new understandings of pathophysiological mechanisms, advancements in monitoring technology, improvements to prognostic evaluation systems, and optimization of therapeutic approaches. The article emphasizes cutting-edge advancements like artificial intelligence-assisted decision-making systems, microcirculation monitoring tools, and precision medicine approaches, which are customized to individual patient needs by combining evidence from basic research and clinical practice. Precision medicine in the cardiac intensive care unit (CICU) is described in this review as a reactive, patient-centered approach that uses real-time clinical, molecular, imaging, and hemodynamic data to customize acute therapeutic and monitoring interventions for critically ill cardiac patients with complicated, frequently multi-organ pathophysiology. Despite tremendous advancements, there are still difficulties in ensuring individualized care and converting these developments into standardized clinical procedures. In order to promote the development of standardized therapy and customized medical practice in this developing field, this thorough overview attempts to provide researchers and doctors with up-to-date information on cardiac intensive care.

## 1. Introduction

With the increasing understanding that conditions like acute coronary syndrome (ACS), cardiogenic shock, and severe arrhythmias require specialized care, the cardiac intensive care unit (CICU) has become an essential part of the management of patients with acute cardiovascular diseases. Since the early 1960s, CICU has undergone constant technological and therapeutic advancements with the goal of enhancing patient outcomes. An important turning point in cardiology was the creation of specialized CICU with the goal of improving the prognosis of myocardial infarction, which has historically been one of the main causes of death globally [1]. CICU has evolved over the years into comprehensive cardiac care facilities that can handle a wider range of urgent conditions, including life-threatening non-cardiac disorders like respiratory failure and septic shock, in addition to cardiac emergencies. This development highlights the significance of CICU in contemporary healthcare systems, where they are essential for handling complicated and varied patient needs.

Patients admitted to CICU are increasingly arriving with a wide range of comorbidities, making their management more challenging as the global population ages and the epidemiology of cardiovascular illnesses changes. The combination of aging populations and the increasing incidence of chronic illnesses calls for a review of current monitoring systems and treatment approaches in CICU. As clinicians work to manage both acute cardiac events and the underlying systemic problems that often accompany these diseases, the necessity for sophisticated monitoring techniques is very evident. Thus, enhancing the quality of treatment for patients with acute cardiovascular problems while simultaneously addressing the complications brought about by various comorbidities is a twin challenge that shapes the current landscape of cardiac critical care [2]. In order to maximize patient care and results, this complexity necessitates a multidisciplinary strategy that integrates several expertise.

Innovative monitoring and treatment tools have been made possible by recent developments in our understanding of the pathophysiology of acute cardiovascular illnesses. Our understanding of cardiac pathophysiology has been enhanced by research ranging from molecular biology to organ-system interactions, which has resulted in advances in therapeutic approaches. The capabilities of the CICU have been greatly improved by the incorporation of new technology, such as sophisticated hemodynamic monitoring systems and mechanical circulatory support equipment. Improving survival rates in critically ill cardiac patients requires more individualized treatment methods and real-time patient status evaluation, both of which are made possible by these advancements [3]. Additionally, the capacity to carry out advanced research in CICU has promoted an atmosphere of ongoing learning and adaptation, allowing physicians to stay up to date on the most recent evidence-based procedures and integrate them into clinical workflows.

Precision medicine in the CICU is a reactive, acute care approach that uses real-time, patient-specific data to customize interventions for critically ill cardiac patients. In order to improve short-term survival and long-term functional results, this strategy focuses on rapid, individualized care while addressing the heterogeneity of CICU patients, who frequently present with acute cardiac episodes and associated multi-organ failure.

The translation of basic research into clinical practice, the development of monitoring and treatment technologies, and the optimization of clinical practices within CICU are the three main dimensions that this review focuses on in order to methodically outline the most recent developments in the field of cardiac intensive care. In addition to highlighting the advancements, the review’s conclusions will point out areas that require more study and improvement in order to improve the effectiveness of cardiac treatment. Our ultimate objective is to contribute to the continuing discussion about the future of cardiac critical care, making sure that it continues to be sensitive to the changing patient care and cardiovascular disease treatment landscape [4].

A thorough literature review was carried out to find peer-reviewed research, reviews, and clinical guidelines on cardiac critical care published between January 2020 and October 2025 across the following databases: PubMed/MEDLINE. Cardiac critical care, hemodynamic monitoring, mechanical circulatory support, artificial intelligence, precision therapy, cardiorenal syndrome, neurocardiac interaction, ferroptosis, exosomes, and prognostic evaluation were among the MeSH terms and free-text keywords that were combined in the search approach. The following criteria was set for inclusion: (1) original research, systematic reviews, meta-analyses, and clinical practice guidelines pertaining to adult and pediatric CICU care; (2) studies that describe new pathophysiological discoveries, technological advancements, or treatment approaches in cardiac critical care. Exclusion criteria included: (1) preclinical research with no promise for clinical translation; (2) single-center case reports with fewer than five patients; and (3) publications that only addressed chronic cardiovascular illness and had no bearing on the CICU.

## 2. New Insights into the Pathophysiological Mechanisms of Cardiac Critical Illness

### 2.1 Molecular Mechanisms of Myocardial Injury and Repair

Novel targets for therapeutic intervention in cardiac critical illness have been revealed by recent developments in cardiovascular research, which have clarified complex molecular mechanisms underlying myocardial injury and healing. A major factor in myocardial damage is mitochondrial dysfunction, which causes cell mortality and contractile dysfunction through decreased oxidative phosphorylation and increased production of reactive oxygen species (ROS) [5]. Notably, activation of inflammasomes that intensify inflammatory cascades inside the heart and ferroptosis, an iron-dependent form of controlled cell death marked by lipid peroxidation, have been found to be important mechanisms aggravating cardiac injury. These findings demonstrate the possibility of reducing cardiac damage by focusing on ferroptosis and mitochondrial pathways.

Simultaneously, the roles of epigenetic changes and the cardiac stem cell niche in myocardial regeneration and repair have drawn attention. Stem cell survival, proliferation, and differentiation are regulated by the milieu surrounding cardiac stem cells, which includes extracellular matrix elements and paracrine signaling. Histone methyltransferases, such as enhancer of zeste homolog 2 (EZH2), are epigenetic regulators that alter gene expression patterns essential for cardiac remodeling and adaptability following damage [6]. In particular, EZH2 demonstrates dual function by functioning as a transcriptional co-activator, coordinating metabolic reprogramming from oxidative phosphorylation to glycolysis during ischemic stress, and suppressing cardioprotective genes through H3K27 trimethylation. This epigenetic flexibility provides opportunities for epigenetic therapeutics and highlights the intricacy of cardiac healing mechanisms.

Additionally, research on cardioprotection has focused on exosome-mediated intercellular communication [7]. Mesenchymal stem cells (MSCs) and other cardiac cells produce exosomes that transport bioactive cargo, such as proteins, lipids, and microRNAs, which alter the activity of recipient cells to support angiogenesis, cytoprotection, and the resolution of inflammation [8]. For example, it has been demonstrated that MSC-derived exosomes can enhance cardiac healing after damage by reprogramming macrophages toward anti-inflammatory phenotypes [9]. Furthermore, it has been shown that exosome-mediated administration of growth differentiation factor-15 (GDF15) reduces fibrosis and improves heart function following myocardial infarction by downregulating fatty acid-binding protein 4 (FABP4) [10]. Exosome-based treatments are positioned by these results as possible translational approaches for heart regeneration.

The function of microRNAs like miR-183-5p and miR-199a-3p in controlling autophagy pathways and cardiomyocyte survival provides more molecular insights. By inhibiting forkhead box O1 (FOXO1), miR-183-5p increases the migration and proliferation of bone marrow mesenchymal stem cells (BMSCs), hence decreasing inflammation and cardiac edema [11]. By altering autophagy and apoptosis, the Early Growth Response 1 (EGR1)/miR-199a-3p/mTOR axis has been linked to worsening myocardial damage under intermittent hypoxia, as seen in obstructive sleep apnea [12]. Potential biomarkers and treatment targets for myocardial damage are provided by these regulatory RNAs [13]. New biomarkers in CICU and their clinical application values can be seen in Table 1.

**Table 1.** **Novel biomarkers in CICU and their clinical utility**.

Biomarker	Molecular target/pathway	Clinical utility in CICU
NGAL	Renal tubular injury	Early detection of AKI in CRS; prediction of CRS mortality
TIMP-2	Renal cell cycle arrest	Early AKI biomarker; risk stratification in post-cardiac arrest AKI
miR-183-5p	FOXO1, BMSCs migration/proliferation	Prediction of myocardial repair after AMI; reduction of cardiac edema
miR-199a-3p	EGR1/mTOR axis, autophagy/apoptosis	Biomarker of myocardial damage in obstructive sleep apnea; risk stratification in ACS
GDF15	FABP4, fibrosis	Reduction of post-AMI fibrosis; guidance of SGLT2i therapy in AMI
FcγRIIa	Platelet activation, cardiovascular events	Prognostic assessment of CICU mortality; prediction of thrombotic events

CICU, cardiac intensive care unit; NGAL, neutrophil gelatinase-associated lipocalin; TIMP-2, tissue inhibitor of metalloproteinases-2; GDF15, growth differentiation factor-15; FcγRIIa, platelet fc gamma RIIa; BMSCs, bone marrow mesenchymal stem cells; EGR1, early growth response 1; AKI, acute kidney injury; CRS, cardiorenal syndrome; AMI, acute myocardial infarction; ACS, acute coronary syndrome; SGLT2i, sodium-glucose cotransporter-2 inhibitors; FOXO1, forkhead box O1; FABP4, fatty acid-binding protein 4.

These molecular processes—ferroptosis, mitochondrial malfunction, epigenetic regulation, and exosome-mediated intercellular communication—combine to form a complex network that controls myocardial damage and repair. To improve outcomes in cardiac critical disease, it is essential to comprehend these pathways in order to create novel regenerative medicine techniques and focused therapies.

### 2.2 Mechanistic Analysis of Hemodynamic Disturbances

A hallmark of cardiac critical disease is hemodynamic instability, and new research has deepened our understanding of how cardiopulmonary and vascular connections lead to multi-organ failure. Since systemic organ failure is caused by abnormalities in this axis, the idea of cardiopulmonary-vascular coupling has evolved [14]. For example, preclinical and clinical models have shown that dysregulated sympathetic activity and inflammatory responses limit organ perfusion in sepsis and cardiogenic shock by affecting cardiac output and vascular tone [15,16].

It is becoming more widely acknowledged that microcirculatory dysfunction plays a crucial role in cardiac critical disease. The vascular barrier is compromised by damage to the endothelial glycocalyx, which leads to tissue edema, capillary leakage, and poor oxygen supply [17]. Myocardial ischemia is made worse by this microvascular damage, which also accelerates the development of heart failure (HF). A vital component of cardiac fluid balance is microvascular integrity. Inflammation-causing pathogenic events, hypoxia, and changes in vascular perfusion and coagulability all upset the delicate balance between capillary filtration and lymphatic fluid evacuation [18]. Interstitial and intracellular water build up as a result of pericyte disintegration and the breakdown of the glycocalyx, which is the primary component of the endothelial filtration barrier.

Developments in systems biology and computational modeling have made it possible to analyze the intricacy of hemodynamic disruptions. Wall shear stress, arterial stiffness, and vascular compliance can all be quantified using patient-specific hemodynamic simulations that include fluid-structure interaction evaluations. Applications of these models to a number of cardiovascular diseases, such as aortic aneurysms and pulmonary arterial hypertension, have shown links between biomechanical forces and the course of the condition [19]. Furthermore, the effects of mechanical circulatory support devices, such as venoarterial extracorporeal membrane oxygenation (VA-ECMO), on cardiac load and output have been clarified by pressure-volume loop analysis and generalized circulatory equilibrium models [20]. This has informed strategies to maximize device use and minimize side effects, such as an increase in left ventricular afterload [21].

For real-time hemodynamic assessment in CICU, noninvasive bedside instruments such as cardiac point-of-care ultrasonography (POCUS) and transthoracic echocardiography have become essential [22]. By facilitating the assessment of pulmonary hypertension, volume status, and biventricular function, these techniques enable customized treatment. To improve risk stratification, new hemodynamic indices like the right ventricular-arterial compliance index (RVACi) have been created to predict right HF following the implantation of a left ventricular assist device [23].

Clinical research has also demonstrated how hemodynamic responses to therapies are dynamic. For example, the effects of continuous positive airway pressure (CPAP) therapy on cardiac output vary according to right ventricular function and pulmonary vascular resistance, highlighting the need for customized care [24,25]. Similar to this, sedation with drugs like propofol can cause notable variations in blood pressure, requiring individualized hemodynamic monitoring.

In coronary artery disease, computational models have become a potent tool for non-invasively monitoring important indicators, offering a good substitute for traditional invasive techniques and enhancing clinical decision-making. While pulsatile simulations might be required to capture complicated dynamics at systole, steady-state simulations are sufficiently accurate and significantly lower computational effort for measures like fractional flow reserve (FFR). This distinction highlights the possibility of using steady-state models selectively in clinical practice, making it easier to integrate computational fluid dynamics modeling by determining when patient care in coronary artery disease can be effectively supported by steady-state simulations’ reduced complexity [26]. These advancements highlight how crucial it is to manage complex circulatory dysfunction and maximize therapeutic interventions in critically ill cardiac patients using a systems-based approach.

### 2.3 Pathophysiological Mechanisms of Advanced Heart Failure in the CICU

Neurohormonal activation and ventricular remodeling are two processes of increasing dysfunction that define advanced heart failure (AHF) and may account for the resistance to conventional therapies. Both the structure and the function of the heart muscle are impacted by these dynamic changes. The sympathetic nervous system (SNS) and the renin-angiotensin-aldosterone axis (RAAS) are two aspects of neurohormonal activation [27]. Conversely, severe left ventricular remodeling involves increased end-systolic volume, dilatation and decreased contractility in response to persistent pressure and volume overload, and hypertrophy. Cardiac output progressively declines when the end-systolic and end-diastolic size of the left ventricle (LV) grow. Increased end-diastolic pressure and pulmonary congestion are caused by impaired LV relaxation.

Ischemic heart disease, hypertension, and other conditions can cause AHF. Heart failure is still mostly caused by ischemic heart disease. The loss of functional myocardium results from both acute myocardial infarction (AMI) and chronic myocardial ischemia. Afterload is increased by chronic hypertension, which leads to LV hypertrophy and ultimately dysfunction [27]. AHF can also be brought on by valve problems. AHF results from volume or pressure overload brought on by severe mitral and aortic valve stenosis or regurgitation.

## 3. Monitoring Technology Innovations and Clinical Applications

### 3.1 Advances in Non-Invasive and Minimally Invasive Monitoring Technologies

Non-invasive and minimally invasive monitoring technologies have advanced significantly in recent years, changing the field of cardiac intensive care by providing continuous, real-time evaluation of vital physiological parameters with less danger and pain for patients. Among these advancements, wearables have become useful supplemental tools that can continuously monitor vital cardiac parameters, such as cardiac output and stroke volume variability. This allows for the early detection of hemodynamic instability, which needs to be confirmed later using gold-standard invasive or non-invasive monitoring techniques [28,29]. These gadgets make use of developments in flexible, small ultrasound transducers, which allow for customized treatment and extended bedside monitoring.

Simultaneously, diaphragmatic and pulmonary ultrasonography methods have been refined to improve volume status evaluation and direct respiratory support choices. For real-time, non-invasive monitoring of changes in pulmonary perfusion, such as those that occur in acute pulmonary embolism, electrical impedance tomography (EIT), especially pulsatility-based EIT, has been validated in animal models. This allows for timely clinical interventions and dynamic insights into ventilation-perfusion matching [30]. Similar to this, tongue sublingual microcirculation microscopy provides direct visualization of microvascular perfusion, providing vital information on tissue oxygenation and perfusion that traditional macrocirculatory parameters might overlook, improving the evaluation of shock states and directing resuscitation efforts.

Continuous glucose monitoring (CGM), a crucial component of managing diabetic patients in critical care, has also advanced thanks to the incorporation of biosensing technologies into wearable platforms. Patient comfort and glycemic control have been enhanced by the transition from invasive finger-prick techniques to minimally invasive and non-invasive biosensors using electrochemical, optical, and electromagnetic principles. Bioimpedance-based wearable rings, which offer continuous glucose assessment with promising accuracy and clinical relevance, and microneedle-based sensors for interstitial fluid analysis are examples of innovations [31,32,33]. Advanced data analytics and calibration methods are used in conjunction with these devices to address issues like motion artifacts and sensor biocompatibility.

Additionally, the arsenal for hemodynamic monitoring has been expanded by the development of non-invasive cardiac output monitors that use electrical biosensing and multi-channel thoracic impedance plethysmography [34,35]. However, current devices need to be further refined to achieve precision comparable to invasive gold standards. Continuous interstitial fluid sampling is made possible by the development of flexible microfluidic chips linked to porous microneedle arrays, which further enable minimally invasive biomarker monitoring for long-term critical care treatment [36].

Wearable and non-invasive monitoring devices have limits, even though they have the potential to revolutionize continuous CICU assessment. A significant clinical issue is alarm fatigue. This raises the possibility of a delayed intervention by desensitizing CICU staff to important alerts. Technical constraints still exist: in patients with severe cardiogenic shock or thoracic pathology, non-invasive cardiac output monitors and wearable ultrasonography devices have an absolute inaccuracy when compared to invasive gold standards.

When taken as a whole, these technological developments in non-invasive and minimally invasive monitoring improve outcomes in cardiac intensive care settings by reducing complications related to invasive procedures and improving early warning capabilities and individualized patient management.

### 3.2 Applications of Artificial Intelligence in Cardiac Intensive Care Monitoring

Artificial intelligence (AI) has quickly emerged as a key element of cardiac intensive care monitoring, transforming the interpretation of intricate physiological data and facilitating clinical decision support and dynamic risk assessment. Deep learning algorithms, especially convolutional neural networks applied to electrocardiogram (ECG) data, have shown remarkable performance in the automated detection of cardiac arrhythmias and prediction of hemodynamic parameters such as left ventricular systolic and diastolic dysfunction [37,38,39]. AI models that are entirely automated and capable of human-like ECG interpretation have been developed, including deep learning convolutional neural networks (CNNs), to examine single, continuous, and intermittent ECG signals. Because these AI systems can recognize minute patterns and signals in the ECG that human interpreters could miss, they are useful non-invasive indicators for cardiovascular diseases [40]. The rapid and accurate detection of issues including arrhythmias, hidden heart diseases, and left ventricular failure is one of the many advantages of using AI in ECG analysis. Physicians may find it useful for diagnosis, interpretation, risk assessment, and sickness management.

AI algorithms have been effectively used to predict electrolyte abnormalities like hyperkalemia, which are linked to higher mortality in patients in the CICU, in addition to arrhythmia identification. Even when laboratory readings are normal, AI-enhanced ECG analysis can detect hyperkalemia, enabling early management and boosting survival rates [41]. Similarly, by combining several clinical factors, AI models have been created to predict acute kidney injury in pediatric CICU patients, allowing for preventative care techniques to lessen renal dysfunction [42].

In order to provide thorough risk profiles and dynamic prognostic assessments, AI merges heterogeneous data sources, such as vital signs, laboratory tests, imaging, and electronic health records, in multimodal data fusion systems. For instance, it has been demonstrated that transformer-based models that integrate textual and tabular data from electronic health records can predict pediatric cardiac arrest more accurately than conventional models, demonstrating the potential of AI to synthesize complex temporal and contextual information [43].

Clinical decision support systems use real-time analysis of large datasets to minimize medical errors, optimize resource allocation, and provide customized treatment recommendations [43]. Implementing AI-guided hemodynamic management techniques within cardiac-improved recovery pathways has been linked to shorter ventilation times and stays in critical care units, showing observable improvements in patient outcomes and healthcare efficiency [44]. Furthermore, automated interpretation and measurement techniques for ultrasound imaging enable quick, precise cardiac and pulmonary evaluations at the patient’s bedside, improving diagnostic capacities in critically ill patients [45].

Although AI-driven monitoring systems interpret physiological data with remarkable precision, their “black box” character continues to be a major obstacle to clinical implementation. Clinicians are reluctant to replace clinical judgment since most deep learning algorithms for ECG analysis and risk prediction do not produce interpretable results. AI is best used as a decision-support tool rather than a stand-alone diagnostic or monitoring modality, even if it can enhance invasive gold standards due to technical and clinical limitations.

Notwithstanding these developments, problems with clinical integration, algorithm validation, and data standardization still exist. To maintain patient safety and physician trust, explainable AI models and ethical issues are essential. However, a new era in cardiac intensive care marked by precision medicine, proactive management, and better patient outcomes is heralded by the merging of AI with cutting-edge monitoring technologies.

## 4. Optimization and innovation of treatment strategies

### 4.1 Mechanical Circulatory Support Technological Innovations

Significant developments in mechanical circulatory support (MCS) have improved outcomes for individuals with severe cardiac failure and increased its applicability. The increased indications and technical improvements of percutaneous ventricular assist devices (pVADs), including the Impella family, are one noteworthy innovation. These devices now provide hemodynamic support with better safety profiles for a wider range of patients, including those undergoing cardiogenic shock and high-risk percutaneous coronary intervention (PCI). Research has shown that compared to low-flow devices, high-flow Impella devices (such as Impella 5.0/5.5) are linked to lower chances of device-related problems like hemolysis and renal damage, which translates into better survival outcomes [46]. Additionally, the therapeutic utility of pVADs has been expanded by their new delivery systems, low-profile full-flow devices with expandable impellers, and downsizing, which have improved their hemodynamic efficacy and ease of insertion [47]. The necessity for optimal patient selection and evidence-based protocols is highlighted by regional variations in pVAD utilization and related costs without evident mortality benefits, despite rising use [48].

Extracorporeal membrane oxygenation (ECMO) in conjunction with left ventricular unloading strategies has demonstrated promise in improving outcomes for individuals with cardiogenic shock [49] concurrently with pVAD development. Although randomized trials have not yet conclusively established mortality benefits, the inclusion of interventional left ventricular unloading during VA-ECMO can improve myocardial recovery and lessen the negative hemodynamic effects of increasing afterload [50]. Critically ill patients can now be transferred and managed in a variety of settings thanks to advancements in ECMO technology, such as mobile ECMO teams and better cannulation techniques, which have increased safety and accessibility [51]. Additionally, new biomaterials are being developed to increase hemocompatibility, decrease thrombogenicity, and increase the longevity of MCS devices, such as hybrid membranes that combine synthetic polymers with decellularized biological tissues [52,53]. A major advancement in pediatric populations is the incorporation of dual-pump systems designed for anatomical and physiological limitations and smaller continuous-flow devices, which are bolstered by new registries that enable data exchange and outcome monitoring [54,55].

Important study design and clinical confounding variables are responsible for the lack of efficacy in randomized controlled trials (RCTs) examining left ventricular unloading during VA-ECMO. First, diverse patient populations with varying degrees of cardiac viability are included in the majority of trials [50]. Furthermore, myocardial stunning and persistent left ventricular distension are linked to delayed commencement of left ventricular unloading (>12 hours after VA-ECMO cannulation), offsetting potential benefits. A combination of short-term and long-term endpoints are used in trials, and some also incorporate non-clinical endpoints that have no bearing on mortality [51]. Meta-analysis and clinical translation are challenging due to this heterogeneity. By stratifying patients based on cardiac viability, standardizing the time of intervention, employing consistent mortality endpoints, and putting in place a basic device maintenance methodology, future RCTs of VA-ECMO unloading must overcome these shortcomings.

The field of cardiac intensive care is changing as a result of these technological advancements in mechanical circulatory support, which range from new biomaterials and ECMO adjuncts to increased pVAD indications and design enhancements. They make it possible to provide patients with severe HF and cardiogenic shock with more accurate, long-lasting, and secure support, highlighting the need for interdisciplinary cooperation and ongoing research to maximize device selection, management techniques, and patient outcomes.

### 4.2 Advances in Precision Pharmacotherapy

Thanks to discoveries from pharmacogenomics, new inotropic drugs, and developing immunomodulatory therapies, precision pharmacotherapy in cardiac critical care has significantly changed. The customized approach to antiplatelet medication, which is especially influenced by genetic profiling of cytochrome P450 2C19 (CYP2C19) variations, is a significant area of advancement. Particularly in patients undergoing PCI or presenting with ACS, tailored antiplatelet regimens based on CYP2C19 genotyping have shown reductions in major adverse cardiovascular and cerebrovascular events without increasing bleeding risk [56,57]. Point-of-care genotyping optimizes P2Y12 inhibitor selection, improves adherence to best practice standards, and speeds up clinical decision-making [58,59]. Additionally, the use of platelet function tests in conjunction with genetic data allows for the dynamic evaluation of medication responsiveness and therapy customization to balance the risks of bleeding and ischemia [60,61]. Precision antiplatelet therapy is widely applicable, as seen by the effective use of these techniques across a variety of groups, including patients with diabetes and various smoking statuses [62,63].

Novel drugs like omecamtiv mecarbil, which directly target cardiac myosin to improve contractility without raising intracellular calcium or myocardial oxygen consumption, represent a paradigm shift in the field of inotropic support. Omecamtiv mecarbil has a safer profile than conventional inotropes and enhances systolic function by extending actomyosin cross-bridge attachment, with selective benefits seen in the ventricular myocardium [64]. Istaroxime, another promising drug, minimizes arrhythmogenic potential and supports blood pressure by combining Na+/K+-ATPase inhibition with sarcoplasmic reticulum Ca(2+)-ATPase (SERCA2a) activation to provide positive inotropic and lusitropic effects [65]. Furthermore, through β1-adrenoceptor pathways, endogenous compounds like 6-nitrodopamine have been found to be powerful positive chronotropic and inotropic agents that provide new treatment targets [66,67].

Immunomodulatory treatments have become popular in the treatment of heart diseases marked by immunological dysregulation and inflammation. In order to minimize cardiac damage and unfavorable remodeling, focused immunosuppressive and immunomodulatory therapies can reduce the complicated immunological activation involved in immunopathogenesis in myocarditis [68]. In a similar vein, immunotherapy strategies are being investigated for ischemic heart disease in an effort to enhance cardiac repair and function by reprogramming immune cells, modifying endothelial function, and reducing fibrosis [69]. With corticosteroids, cytokine inhibitors, and other medications used to reduce hyperinflammatory states and enhance outcomes, the COVID-19 pandemic has further highlighted the significance of immunomodulation [70,71]. Innovative platforms for precise immunomodulation are made possible by developments in functional DNA materials and nanomedicine, which allow for targeted delivery and regulated immune responses in oncologic and cardiovascular illnesses [72,73].

Innovative inotropes with better safety profiles, advanced immunomodulatory techniques, and genetically directed antiplatelet regimens are all integrated into precision pharmacology in cardiac critical care. These developments have the potential to improve outcomes for critically ill cardiac patients by increasing medication efficacy, lowering adverse events, and personalizing care.

## 5. Multi-Organ Function Support Strategies

### 5.1 Comprehensive Management of Cardiorenal Syndrome

A complicated interaction between cardiac and renal dysfunction is known as cardiorenal syndrome (CRS), in which impairment in one organ causes or exacerbates malfunction in the other. In patients with acute and chronic HF worsened by renal illness, this reciprocal interaction is becoming increasingly well acknowledged as a crucial factor in determining morbidity and death [74]. Hemodynamic changes, such as decreased renal perfusion as a result of lower cardiac output, neurohormonal activation, including the renin-angiotensin-aldosterone system, oxidative stress, inflammation, and cytokine release, are all part of the pathophysiology of CRS. Multiorgan failure is typical in systemic diseases such as sepsis and COVID-19, where these pathways are most prominent [74]. For prompt care, early detection of acute kidney injury (AKI) in CRS is essential. In contrast to conventional markers like serum creatinine, which frequently lag behind real injury, novel biomarkers like neutrophil gelatinase-associated lipocalin (NGAL) and tissue inhibitor of metalloproteinases-2 (TIMP-2) have emerged as sensitive indicators for early renal injury [75,76]. These biomarkers make it easier to identify subclinical AKI and allow for prognostication, which directs treatment choices.

Continuous renal replacement therapy (CRRT) is still essential for treating severe AKI in patients with CRS, especially those who are hemodynamically unstable. To improve patient results, CRRT time and dosage must be optimized. Research indicates that early CRRT, customized to each patient’s hemodynamic status and renal function, can promote fluid balance, slow the progression of renal impairment, and lessen cardiac congestion [77,78]. The best time to start is still debatable, though, as some research shows that early commencement does not significantly reduce mortality [78,79]. This highlights the importance of individualized evaluation. Effective solute clearance and hemodynamic stability must be balanced in the dosing method, which includes blood flow rates and ultrafiltration volumes [80]. Additionally, multidisciplinary cardiorenal programs that incorporate cardiologists, nephrologists, pharmacists, and nursing personnel have shown promise in providing all-encompassing care, enhancing diagnostic precision, and optimizing treatment plans [81,82].

Computational ecosystems that include multi-omics and clinical data have improved mechanistic insights into heart-kidney interplay, providing a comprehensive picture of CRS pathogenesis [83]. These methods could make it possible to find new therapeutic targets and customized treatment plans. Furthermore, point-of-care ultrasound, such as renal Doppler and venous excess ultrasound scoring, has become indispensable for evaluating renal perfusion, right heart function, and volume status, directing fluid management, and initiating CRRT [84,85]. The potential of biomarkers indicating oxidative stress, fibrosis, and inflammation to improve CRS diagnosis and prognosis is also being studied [86]. Additionally, sodium-glucose cotransporter-2 inhibitors (SGLT2i) have demonstrated positive effects in improving renal outcomes and lowering hospitalization for HF [87,88]. These benefits are probably due to enhanced tubuloglomerular feedback, diuresis, and decreased renal oxygen consumption. However, CRS patients are frequently underrepresented in randomized controlled trials, which highlights the need for focused studies in this population and necessitates extrapolation from HF and chronic kidney disease (CKD) studies [74].

We suggest focused trial designs and registry-based strategies that use real-world data and practical study techniques to produce generalizable findings in order to address the underrepresentation of CRS patients in therapeutic trials. Pragmatic multicenter CRS-specific RCTs: these studies should use pragmatic endpoints instead of surrogate endpoints and include consecutive CICU patients with CRS [74]. CRS nested sub-studies: to enroll CRS patients without the requirement for independent trial enrollment, incorporate CRS-specific sub-studies into sizable, ongoing heart failure and AKI trials. These sub-studies can evaluate the effectiveness of therapies for CRS-specific outcomes and subgroups [88]. These methods produce evidence that is immediately applicable to CICU clinical practice while addressing the fundamental issues with CRS trial recruitment.

Integrated multidisciplinary treatment, optimal CRRT timing and dose, early identification using novel biomarkers, and integration of developing therapies are all necessary for comprehensive therapy of CRS. With the goal of improving patient outcomes through individualized and focused therapy, developments in biomarker research, computational modeling, and diagnostic imaging continue to increase our knowledge of, and approach to, treating this complicated illness.

### 5.2 Neurocardiac Interaction and Brain Protection

The pathophysiology of brain injury linked to cardiac critical disease is significantly influenced by the complex neurocardiac axis. One important pathogenic mechanism that underlies brain damage associated with cardiac arrest and other neurocardiac disorders is autonomic nervous system dysfunction [89]. Impaired brain perfusion, neuroinflammation, and subsequent neuronal damage result from disruption of autonomic balance after cardiac events. This autonomic dysfunction has been associated with negative neurological consequences and manifests as altered heart rate variability [90]. Furthermore, although their long-term clinical implications are still unclear, critical illness-associated brain microbleeds, especially in the corpus callosum, have been found to be radiographic indicators of hypoxic-ischemic injury following cardiac arrest [91].

In order to reduce ischemia-reperfusion injury after cardiac arrest and in other acute encephalopathies, targeted temperature management (TTM) has emerged as a proven neuroprotective technique. TTM improves neurological outcomes and lowers mortality by lowering excitotoxicity, regulating metabolic demand, and attenuating inflammatory cascades [92,93]. It is crucial to optimize TTM methods, including when to reach the target temperature and how long hypothermia lasts. Reduced neurological consequences have been linked to the early onset and maintenance of normothermia or moderate hypothermia (32–36 °C) [94,95]. TTM and quick cooling techniques are equally important in preventing multiorgan dysfunction and neurological damage in heat stroke, which shares pathophysiological characteristics with hyperthermia-induced brain injury [96,97]. In juvenile hemorrhagic shock and encephalopathy syndrome, there is growing evidence to support the use of mild cerebral hypothermia followed by targeted temperature regulation to enhance neurological outcomes [98].

During the crucial post-resuscitation phase, continuous neuromonitoring methods—such as near-infrared spectroscopy to measure cerebral oxygen saturation—offer important insights into the state of cerebral perfusion. The significance of cerebral oxygen monitoring in directing therapeutic therapies is highlighted by the correlation between reduced cerebral oxygenation and long-term cognitive impairment [99]. Furthermore, somatosensory evoked potentials and other evoked potentials are trustworthy instruments for early neuroprognostication during cardiac arrest, supporting clinical judgment on whether to continue or stop life-sustaining treatments [100].

The importance of microglia-mediated neuroinflammation in cardiovascular illnesses has also been highlighted by advances in our understanding of neurocardiac interactions, indicating that neuroimmune regulation may offer a new treatment approach [101]. Furthermore, autonomic dysfunction identified by heart rate variability studies in individuals with neurotrauma and rapid eye movement sleep behavior disorder suggests wider consequences of neurocardiac dysregulation in neurodegenerative processes [90].

With molecular connections to autonomic nervous system function, cerebral perfusion, and neuroinflammation, CICU-specific therapies directly alter the neurocardiac axis and impact neurological outcomes. In CICU patients experiencing cardiac arrest, α2-adrenoceptor agonists increase cerebral perfusion and decrease neuroinflammation by lowering sympathetic hyperactivity and maintaining autonomic balance [16]. Although severe left ventricular unloading can lower coronary perfusion pressure and raise the risk of cerebral hypoperfusion, VA-ECMO and pVADs enhance cardiac output and cerebral perfusion [20]. Neurological damage is less likely when MCS flow protocols are standardized [99]. Improving neurological outcomes in critically ill cardiac patients requires the explicit incorporation of neurocardiac monitoring into CICU intervention procedures.

**Fig. 1.**
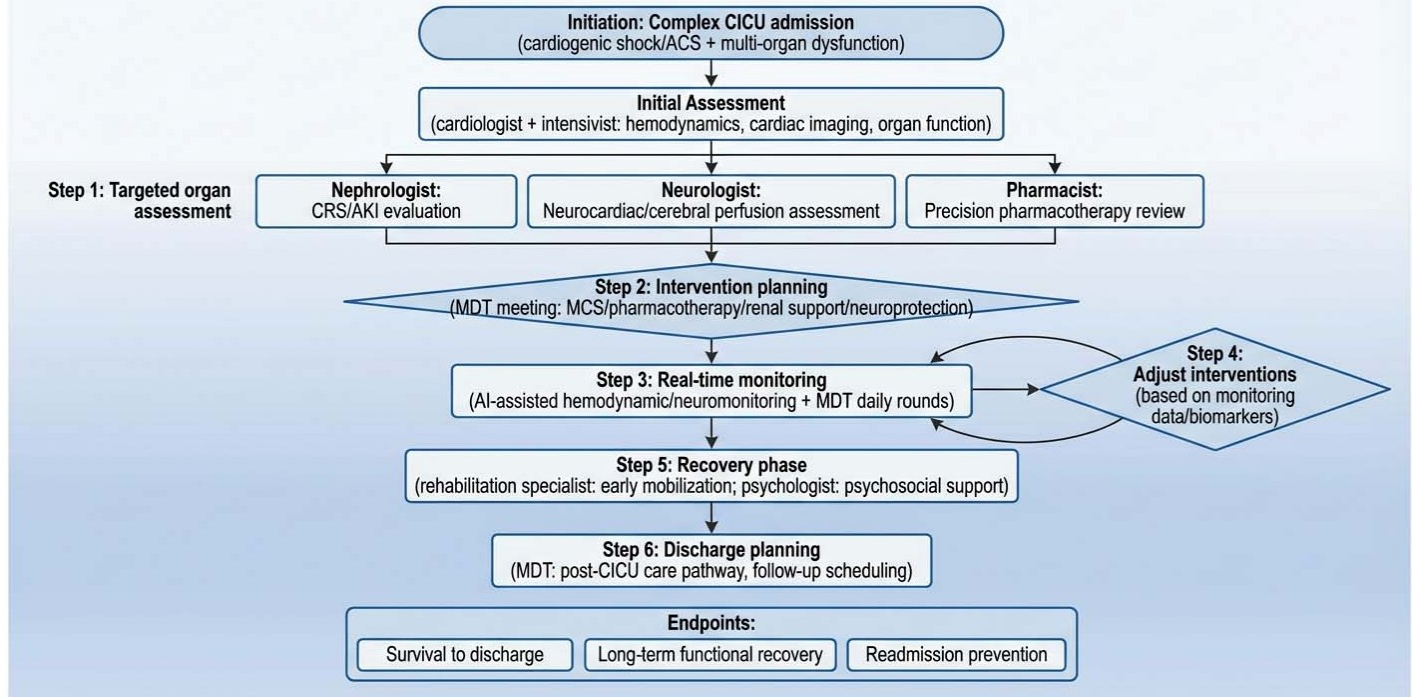
**Multidisciplinary management algorithm for complex CICU patients of an integrated care pathway involving cardiologists, intensivists, nephrologists, neurologists, pharmacists, and rehabilitation specialists**. CICU, cardiac intensive care unit; ACS, acute coronary syndrome; CRS, cardiorenal syndrome; AKI, acute kidney injury; MDT, multidisciplinary team; MCS, mechanical circulatory support; AI, artificial intelligence.

In clinical practice, it is crucial to combine TTM with all-encompassing supportive care, which includes managing ventilation, hemodynamics, and metabolic disorders. Fig. 1 illustrates the multidisciplinary management algorithm for patients in complex CICU. Although their use necessitates careful consideration of time, anticoagulation, and potential consequences, emerging technologies like ECMO paired with CRRT offer multi-organ support in severe situations [102,103]. Additionally, advances in therapeutic medication monitoring during extracorporeal treatments provide the best possible pharmacokinetic and pharmacodynamic care for patients in critical condition [104].

Brain injury outcomes in cardiac critical illness are greatly influenced by neurocardiac interactions. Brain protection is based on targeted temperature control, ongoing neuromonitoring, and coordinated multi-organ support techniques. Neuroprognostication and therapy efficacy will be improved by ongoing research into neuroimmune systems and cutting-edge supportive technology, which will ultimately improve neurological recovery and quality of life for critically sick patients.

## 6. Prognosis Assessment and Rehabilitation System

### 6.1 Risk Prediction Model Updates

With the incorporation of clinical indicators, biomarkers, and imaging data into machine learning models, the prognostic evaluation of critically ill cardiac patients has changed dramatically, improving risk stratification accuracy. Conventional static risk scores, such those for liver failure [105], alcoholic hepatitis [106], and pulmonary thromboembolism [107], have offered fundamental prognostic tools but frequently lack dynamic flexibility and thorough integration of multimodal data. In order to produce more accurate and customized risk forecasts, recent developments in machine learning have made it possible to create models that integrate heterogeneous data sources, such as vital signs, laboratory biomarkers, and imaging features. By including a wider range of clinical variables while maintaining data privacy, federated learning techniques, for instance, have demonstrated greater performance over traditional indices like pulmonary embolism severity index (PESI) and simplified pulmonary embolism severity index (sPESI) when applied to acute pulmonary thromboembolism [107]. Similarly, platelet Fc gamma receptor IIa (FcgammaRIIa) expression, a new biomarker linked to cardiovascular events, has been quantified using machine learning algorithms, yielding precise and repeatable predictive data [108]. Significantly, prognostic estimations may now be updated in real-time as patient data changes thanks to the development of dynamic risk assessment tools, which enable prompt therapy modifications. The potential for ongoing risk monitoring in clinical settings is demonstrated by the dynamic Bayesian network models employed in risk assessment of circumstances like marine oil spills [109] and yachting tourism safety [110]. Furthermore, risk models are increasingly incorporating quality of life indicators and long-term functional prognosis, realizing that patient-centered outcomes cannot be fully captured by survival alone [111]. For example, extended recovery durations of left ventricular ejection fraction during hospitalization have been found to be independent predictors of reduced cardiac function after discharge in patients with fulminant myocarditis [112]. These all-inclusive models highlight the trend toward personalized medicine, where risk prediction is not only more precise but also dynamically responsive to patient status changes, ultimately enabling improved, customized care pathways in CICU.

### 6.2 New Concepts in Cardiac Rehabilitation

In order to improve patient outcomes and quality of life, cardiac rehabilitation (CR) has experienced revolutionary advancements, including early commencement, multidisciplinary approaches, and the utilization of technology. Ultra-early rehabilitation therapies, including mobilization in CICU, have been demonstrated to shorten hospital stays without sacrificing safety, and there is growing evidence to support their safety and effectiveness [113]. When compared to traditional programs, hybrid CR models that combine synchronous tele-rehabilitation at home with center-based supervised sessions have shown noninferior improvements in functional capacity, addressing accessibility issues and disruptions caused by pandemics [114,115]. Patient adherence and efficacy have been further enhanced by remote monitoring-guided home rehabilitation schemes, especially when psychological support and cognitive training components are added [116]. By reducing depression symptoms, improving cognitive function, and encouraging lifestyle changes, the incorporation of psychological therapies into all-encompassing rehabilitation regimens is becoming more widely acknowledged for its role in improving overall prognosis [117,118]. Notably, gender differences in CR participation have been lessened and psychological results have improved with intensive cardiac rehabilitation (ICR), which includes non-exercise components including stress management and peer support [118]. In order to improve exercise capacity and quality of life in patients with cardiovascular disease, resistance training is increasingly advised within CR to address common lean mass anomalies and sarcopenia [119]. Additionally, a comprehensive evaluation of the psychological well-being of patients undergoing CR is being conducted, with customized interventions created to meet sociocultural aspects and individual requirements [120]. Together, these developments signify a paradigm shift away from traditional CR and toward a comprehensive, patient-centered rehabilitation framework that maximizes recovery and long-term cardiovascular health by including early mobilization, remote technologies, and psychosocial support.

## 7. Conclusion

**Fig. 2.**
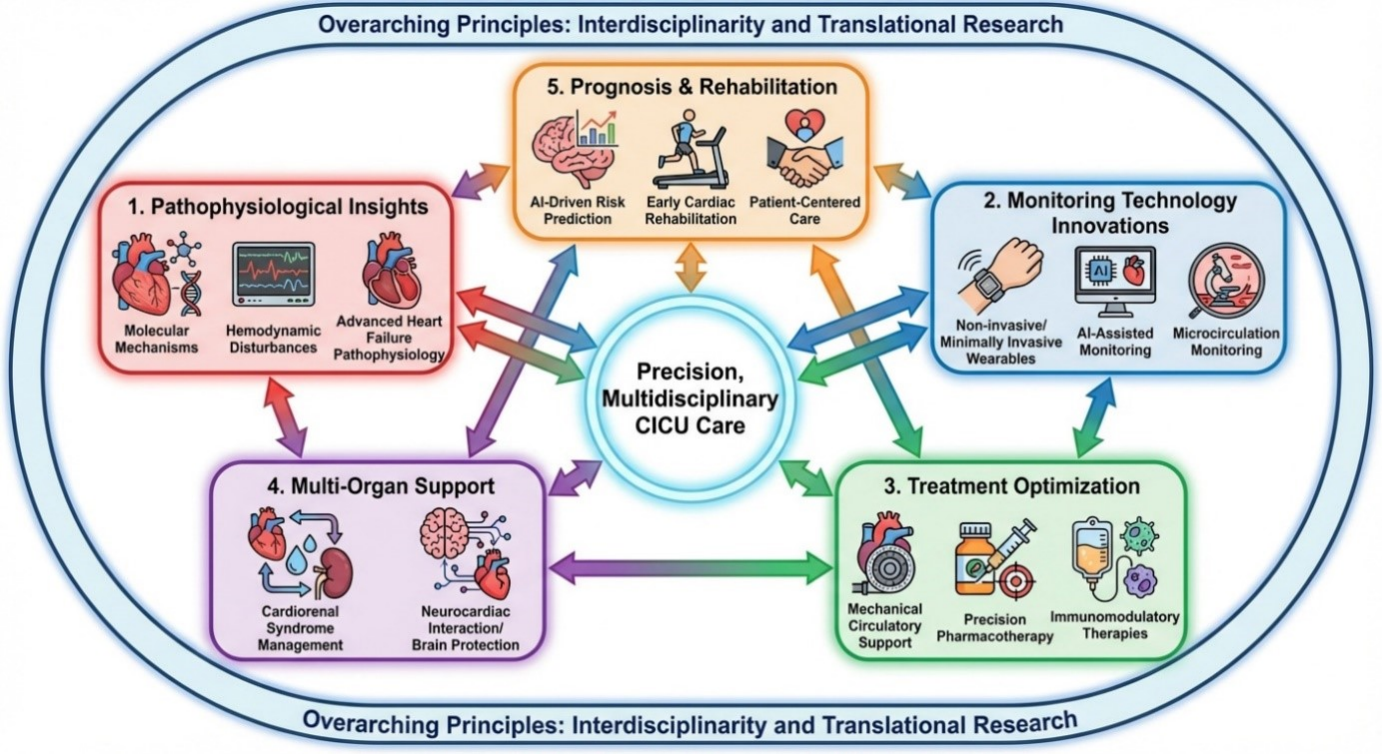
**New insights into cardiac intensive care: core advancements and interconnections, including the five key thematic areas of pathophysiological insights, monitoring technology innovations, treatment optimization, multi-organ support, prognosis/rehabilitation**. CICU, cardiac intensive care unit; AI, artificial intelligence.

The field of cardiac intensive care has seen tremendous progress in the comprehension of pathophysiological mechanisms, monitoring technologies, and therapeutic interventions, all of which have contributed to the evolution of diagnostic and treatment paradigms toward increased accuracy and customization. The core advancements and interrelationships in CICU in Fig. 2. According to experts, these advancements highlight how crucial it is to combine interdisciplinary cooperation, technical innovation, and translational basic research in order to advance the profession. The use of state-of-the-art tools like AI is an example of how developing technology can improve patient monitoring, risk assessment, and individualized treatment planning.

A dynamic environment where mechanistic insights into heart pathology must be balanced with practical clinical applications is revealed by balancing various research viewpoints and discoveries. For example, whereas novel biomarkers and organ interaction pathways present exciting opportunities for targeted therapy and early detection, their therapeutic value requires thorough validation using large-scale, real-world data and longitudinal follow-up investigations.

Despite revolutionary advancements in cardiac critical care, there are three main obstacles that prevent these developments from being translated into uniform clinical practices. These obstacles are consistent with the diversity of CICU patient populations and healthcare systems around the world. First, adoption of cutting-edge technology is limited in healthcare settings with low and intermediate incomes, as well as in high-income centers with limited resources, due to their high initial and ongoing expenses. Second, CICU clinicians, nurses, and pharmacists need specialized training to implement novel therapies. Lastly, reimbursement rules do not currently cover these procedures, and many CICU innovations do not have official regulatory approval for acute cardiac care. To overcome these obstacles, multicenter cooperation is needed to create flexible, tiered clinical protocols. Additionally, advocacy for regulatory simplification and reimbursement reform for evidence-based CICU innovations is necessary.

Future research should focus on clarifying the intricate interactions between the cardiac and extracardiac organ systems, which frequently determine patient outcomes in critical care environments. Simultaneously, it is necessary to optimize therapy approaches and create new biomarkers with a focus on improving specificity and efficacy. In order to continuously improve therapeutic practices based on long-term patient outcomes, the methodical gathering and analysis of real-world data will be crucial.

To improve the general quality of cardiac critical care, it is also crucial to create multicenter collaboration networks and standardize quality assessment systems. These programs will expedite the conversion of research findings into clinical guidelines, encourage uniformity in the provision of care, and facilitate knowledge exchange. The ultimate goal of these initiatives is to enhance the quality of life and survival rates of people with serious heart diseases.

In summary, the development of cardiac intensive care shows a careful balancing act between cutting-edge research and practical clinical application. The area is well-positioned to achieve significant gains in patient prognosis and care tailoring by adopting multidisciplinary approaches, utilizing technology breakthroughs, and dedicating itself to rigorous data-driven evaluation. The future of cardiac critical care will continue to be shaped by this all-encompassing and integrative approach, which guarantees that treatment interventions are both scientifically supported and customized to each patient’s unique needs.
